# Hernia in Children
*A Clinical Lecture delivered at St. Bartholomew's Hospital.


**Published:** 1906-04-14

**Authors:** D'Arcy Power

**Affiliations:** Surgeon and Lecturer on Surgery at St. Bartholomew's Hospital; Surgeon to the Bolingbroke Hospital; Senior Surgeon to the Victoria Hospital for Children, Chelsea.


### Hospital Clinics.

### HERNIA IN CHILDREN.*

By D'Arcy Power, F.R.C.S.Eng., Surgeon and Lecturer on Surgery at St. Bartholomew's Hospital; Surgeon to the Bolingbroke Hospital; Senior Surgeon to the Victoria Hospital for Children, Chelsea.

Everything connected with hernia in childhood is of the greatest interest to the surgeon on account of the frequency with which he is consulted about the affection. It occurs in the children of the poor rather more often than in those of the well-to-do, and in each class it offers its own problems. Rupture in a pauper child precludes a livelihood from mechanical toil, often the only means of subsistence available when it grows up; whilst it renders the son of wealthy parents morbidly self-conscious, prevents him from making the best use of his education at a public school, and finally shuts him out of the public services for which his talents might otherwise peculiarly fit him. I shall not be wasting your time, therefore, to-day by asking you to consider the subject of hernia and its treatment in childhood.

### Umbilical Hernia.

There are really only two forms of hernia in children—umbilical and inguinal—and as umbilical is the less important we will take it first. Femoral and diaphragmatic herniæ are too rare to merit much consideration in children. Umbilical hernia occurs in two forms, the one congenital and a true exomphalos, the other acquired and a protrusion of the navel. In exomphalos the tumour is either embryonal or fœtal, and is formed by a dilatation of the structures forming the proximal part of the cord. The sac is generally fusiform in shape, and is so transparent that the coils of intestine are visible through it when the child is born. The cord itself often shows evidence of degenerative changes in Wharton's jelly, as it has cysts upon it filled with semi-solid material. Exomphalos, too, may be associated with other evidence of defective development, such as spina bifida and congenital lordosis. I show you a specimen which was sent to me a few years ago by Mr. J. J. De Z. Marshall. It is a good example of a congenital umbilical hernia, and is preserved in the museum as No. 2,156. It is thus described in the catalogue:—

"The sac of a congenital umbilical hernia. The tumour appears to be formed by a dilatation of the coverings of that portion of the umbilical cord which was nearest the body of the child. It is fusiform in shape, and has the main constituents of the funis running as a bundle along its lower border. The wall of the sac consists of a thin and soft membrane, which was so transparent, whilst the specimen was fresh, that the coils of intestine could readily be seen within it. The external surface was polished and closely resembled the outer surface of the cord, whilst internally the sac is covered with a smooth layer which is apparently derived from the peritoneum. The funis reappears at the apex of the tumour, and has on its under surface a cyst containing a viscid fluid. The specimen was obtained from a newly-born child at full term. The labour was quite natural, but a placenta-like mass was attached to the child's abdomen. On closer examination, with the aid of a candle, this mass was found to be a transparent sac, containing several coils of small intestine, measuring in all about a foot in length. The sac was situated in the funis at the part nearest to the child's abdomen. The cord was ligatured and divided in the ordinary manner, and an ineffectual attempt was made to get the bowel through the umbilical aperture. The sac was then carefully opened on a director, and after division of the umbilical ring the intestine was returned into the abdominal cavity. The child survived for three days, and died of peritonitis."

I fear that this is the usual ending of exomphalos. I have had two or three cases under my care at different times, but they have all died. Still it is well worth while to attempt an immediate operation to replace the intestine and suture the abdominal wall, for by so doing you will occasionally save a life which would otherwise undoubtedly perish.

An acquired umbilical hernia is found with equal frequency in males and females. It is very common, and is caused by anything which leads the child to strain. The more usual causes, therefore, are repeated vomiting, constant crying, or coughing—especially from whooping-cough—phimosis, constipation, and diarrhœa. It is sometimes seen in rickety children who have much abdominal distension, and if no cause is obvious at first sight it is well to make a rectal examination, for I have known cases where it has been associated with a rectal polypus. "Starting of the navel" is a bugbear to many mothers, but a good prognosis may always be given. It is invariably cured before puberty, and it does not lead to an umbilical hernia later in life. The treatment consists in removing as far as possible any cause which makes the child strain. The local treatment lies in closing the aperture in the abdominal wall and keeping it in apposition for a considerable length of time. The best means of doing this is to sew a penny in a couple of layers of lint. The child is then placed flat upon its back, the protrusion is reduced, and the pad is applied from below upwards so as to convert the umbilical aperture into a slit. The bowel is thus prevented from escaping, and the pad is kept in place by two strips of waterproof strapping placed over it crosswise. A broad flannel bandage is sewn round the abdomen, and the pad and bandage are changed by the surgeon as often as may be necessary, care being taken on each occasion that the rupture does not protrude. In a very few cases a radical operation may be necessary, if, in spite of all treatment, the hernia continues to increase in size. The operation consists in refreshing the edge of the ring and suturing it transversely. I have never had to open the peritoneum or remove the sac.

### Inguinal Hernia.

There are several points in connection with the clinical aspects which make inguinal hernia in

* A Clinical Lecture delivered at St. Bartholomew's Hospital.

children interesting. It should be remembered that, owing to anatomical peculiarities, the hernia in a child is situated higher in the abdominal wall than is usual in adults, and that it has a somewhat less tendency to become scrotal. A hernia, too, which is perfectly obvious at one time may wholly disappear at another time; nor can it always be made to reappear when the child cries or strains. You must exercise some caution, therefore, in making a diagnosis, and not say that no hernia exists because you are unable to see it and because the abdominal ring feels normal, especially if the mother tells you that the child has a swelling in the inguinal ring which varies in size from time to time. The presence of a small hernia may be masked by the coexistence of an encysted hydrocele of the processus vaginalis or by an undescended testis, and the surgeon should therefore make a routine of examination of the scrotum and assure himself that both testicles are in it in every case that he is called upon to examine. He will thus avoid many pitfalls, and will sometimes be led to recognise an interstitial hernia and sometimes a hernia in connection with an undescended testis which he might otherwise have overlooked. When I watch you examine a baby, supposed to be ruptured, it always seems to me that you take for granted that both testicles are in the scrotum, whilst, as a result of practice, it is one of the first things that I satisfy myself about. It would be well if you acquired the same habit.

When you have satisfied yourself that you have an uncomplicated hernia to deal with the first question put to you by the mother will be whether an operation is necessary or whether a truss will not be sufficient. Several points must be taken into consideration before you answer. First, and applicable to every child, is the age. I think that in every case nature should be given a chance of curing a simple inguinal hernia. Considerable changes take place in the inguinal canal within a few months of a child's birth. The inguinal canal hardly exists in a new-born baby. The external and internal abdominal rings are nearly opposite to each other, and the rings themselves are proportionately larger than they are in adults; the external ring, too, is situated higher in the abdomen in children, so that a scrotal hernia is less common. The pelvis slowly increases in size as the child grows, the inguinal canal becomes longer and more oblique, whilst the external ring approaches the top of the scrotum, and the rings themselves become relatively smaller. It is well, therefore, to await these changes and to allow the baby to wear a wool truss at any rate for the first six months of its life. If it be well applied by a careful mother or intelligent nurse the truss is sufficient to prevent the rupture coming down, it is easily kept clean, and it is so soft that it does not exert any injurious pressure upon the skin. But it must be clearly understood that nature effects a cure and not the truss, and that the hernia must be absolutely prevented from entering the sac, or it cannot be cured. This form of truss, therefore, is, as a rule, only useful in private practice, for the mothers who bring their children to the out-patient rooms of hospitals are unable to employ it properly. If the hernia still descends when the wool truss is worn, and in any case at the end of six months the wool truss should be replaced by the ordinary spring truss covered with india-rubber to make it washable, and this truss should be worn until the child is three or four years old.

The tissues are so delicate, the pillars of the ring are so weak, and the difficulties of securing asepsis in infants are so great, that I dislike operating on babies as a routine proceeding for the cure of hernia until they have attained a reasonable size. There are, however, exceptions to the rule that a radical operation should be deferred until a child is three or four years old, and these exceptions will be considered presently. There are many ways of performing a radical operation for the cure of hernia in children. They agree in the single detail of isolating the sac, but in all other points they differ with the practice of the individual surgeon. For my own part I isolate the sac and ligature it as near as possible to the internal abdominal ring, first assuring myself that the sac is empty. I then cut away the sac on the distal side of the ligature and secure the stump to the inner wall of the peritoneum above the internal ring by passing the ends of the ligature through the whole thickness of the abdominal wall except the skin and superficial fascia. The ends of the ligature are passed separately through the wall by threading them on a nævus needle, and the two ends are tied together and cut short. The pillars of the ring are then closed with silk sutures, which I generally pass in front of the spermatic cord and not beneath it as is the practice when operating upon an adult.

A very large proportion of the cases of inguinal hernia occurring in children are congenital hernia. I cannot give you the exact number, but my idea is that about three out of every five cases upon which I perform the radical operation are congenital herniæ. In many cases, therefore, it is impossible to isolate the sac because it forms a part of the tunica vaginalis which has remained patent. It is then necessary to make a new tunica vaginalis by dividing the sac transversely after it has been partially isolated and separated from the constituents of the spermatic cord which are closely attached to its posterior wall. The isolation of the sac in these cases should always be commenced as near the external abdominal ring as possible, care being taken, of course, not to mistake the processus vaginalis for the hernial sac lining it, because if this mistake be made the spermatic cord will necessarily be ligatured and divided at a later stage in the operation.

*Contents of the Sac.*—The sac is usually empty in a child who has been properly prepared for a radical operation, but if it has strained much under the anæsthetic a loop of small intestine may be present in the sac, which must be returned into the abdomen before it is ligatured and the ring is closed. Small tags of omentum are often found in the sac, sometimes lying free and sometimes attached by slight adhesions. In accordance with the teaching of Mr. Kellock I always treat these omental protrusions with the greatest care, for they seem to be a factor in recurrence after operation if they are merely pushed back into the abdomen or are tied off with the sac. It is my rule, therefore, to isolate them in every case and pull them down until they can be ligatured so high up that the stump after removal lies well above the internal abdominal ring.

Irreducible herniæ are rare in children, but incarcerated herniæ where the intestine, on account of its size, cannot be returned into the abdominal cavity are not very uncommon. They occur chiefly on the right side, and are nearly always herniæ of the cæcum. I had a good instance of the manner in which a cæcocele is a cause of irreducibility a short time since. A boy came with an irreducible hernia on the left side, and I thought at any rate this could not be due to the cæcum. But as soon as I had cut down upon it I found that the viscera were transposed and the hernia consisted of the cæcum and its appendix.

Cæcoceles, on the other hand, are sometimes too easily reduced, and return with equal facility owing to the large size of the opening in the abdominal wall through which they protrude. I believe that nearly all the large and easily reduced scrotal herniæ which occur on the right side in babies a few weeks old are herniæ of the cæcum. I always dread operating upon this form of hernia because they are associated in my mind with dirty and shiftless mothers, neglected children, uncontrollable ruptures, and only too often unsatisfactory results. But in spite of these drawbacks large scrotal herniæ need operation at an earlier age than any other form, and as a matter of urgency rather than of discretion. They cannot be kept in place by a truss, they are always in the scrotum, and they tend to become larger.

Twice lately I have found the urinary bladder forming a hernia; both patients were females—one aged 12 and the other 26—and in both the rupture was on the right side. Neither patient had complained of any trouble in micturition, nor had either noticed any variation in the size of the hernia in relation to the passage of water. The elder girl had only noticed the presence of a hernia for a few months before she underwent an operation.

*Strangulation.*—Strangulation of a hernia in a young child is of rare occurrence when compared with the very large number of children who suffer from hernia. This is due to the greater elasticity of the tendinous and fibrous structures in children. C. Stern [1] says that the proportion of cases occurring in children as compared with adults is as 1 to 108. About one hundred operations for the relief of strangulated herniæ have been recorded, and of these three have been under my care. Contrary to what might have been expected, the mortality is by no means high in those cases in which herniotomy has been performed. About sixteen cases have been submitted to operation during the first month, and about as many in the second. Nearly half the cases in children under a year old are said to occur within the first three months. My earliest case in point of years was a premature child, aged five weeks, who was admitted into St. Bartholomew's Hospital under my care on April 15th, 1901. The boy was brought with a history that a swelling was first noticed in the left groin on the 12th, and that on the 13th he cried all day, and vomited every time he was put to the breast. The vomiting continued until the time of his admission to the hospital, and the bowels had not been open since the 12th, at 9 A.M. The child had been born prematurely at seven months. He was blue, and collapsed when I saw him, and in the left inguinal region there was a hernia as big as a walnut. The hernia was cut down upon as taxis under chloroform failed to reduce it. The sac contained a small piece of omentum and a knuckle of small intestine rather deeply congested. The hernia was of the congenital variety, and there was no fluid in the sac. The constriction was situated at the internal ring, and was very tight. The stricture was divided, the bowel was returned, and the sac was isolated, ligatured in two places, and divided. The distal part was left to form a tunica vaginalis, whilst the proximal portion was attached to the peritoneal surface of the abdominal wall in the usual manner. The external abdominal ring was closed with a single suture, and the edges of the wound were united with silkworm gut. The child bore the operation well, and was fed by the mouth every four hours from the time that he recovered from the anæsthetic. The bowels were open on the following morning, and he never vomited. The wound was perfectly healed on the fourth day, when the sutures were taken out, and the child left the hospital on April 30th, fifteen days after its admission. But mark the ingratitude of the parents and the act of Nemesis. On May 21st his mother brought him to the Metropolitan Dispensary, where I then acted as surgeon, for a hernia on the right side. By bad luck she came on my day of attendance. I recognised the scar on the left side, and as the child had a tight phimosis to which the right rupture seemed attributable I circumcised him, and he again recovered. He thus passed through two surgical operations within eight months of the time when he was conceived.

[1] Cbl. f. Chirurgie, 1894.

The second case was that of a child aged ten months and a half, who was said to have strained himself whilst coughing violently at three o'clock in the afternoon. When I saw him eight and a half hours after the onset of symptoms I found that he had a tumour of about the size of a hen's egg which occupied the scrotum and the inguinal region on the right side. The swelling possessed the ordinary signs of strangulated hernia, except that it was perfectly translucent. The child did not seem to be in much pain; he had not vomited, and had taken his food as usual; the bowels had been open twice since the strangulation occurred. The pelvis and abdomen had been elevated, and taxis had been tried unsuccessfully. I exposed the sac, and on opening it some clear fluid escaped and the gut was found to be congested. The stricture was situated at the neck of the sac: it was fairly tight, but was divided without difficulty or injury to the surrounding parts. The bowel was then returned, and the usual operation for radical cure was performed. The child made an uninterrupted recovery, and left the hospital within three weeks of his admission.

The next case is interesting because it has a very complete history from the first protrusion of a piece of small intestine which became irreducible, then congested, and finally caused the ordinary symptoms of strangulated hernia. The differences, too, between the tissues of a child and an adult are well defined: first by the comparatively slight amount of congestion of the bowel which followed upon its constriction; and secondly by the fact that the greater extensibility of the tissues allowed the inguinal canal to be dilated until the bowel could be returned without any division at the seat of constriction. A boy was brought to the hospital on the morning of January 16, aged 2½ years, with an irreducible tumour in the right groin. The mother said that she had first noticed the lump three weeks before, but the child had complained of pain at the bottom of his stomach for some months. On January 15th he had felt more pain, and his parents said that the lump was larger than it had been, and could no longer be put back. During the 24 hours previous to his admission to the hospital the child had refused all food, and he had vomited incessantly throughout the night. When I saw the child he lay in bed with his right leg drawn up and with a fairly tense tumour about the size of a plover's egg in his right groin. I failed to reduce the swelling by gentle taxis under an anæsthetic, and I therefore exposed the sac. The tumour proved to be an inguinal hernia containing a loop of small intestine. The constriction was tight, but the bowel was only slightly congested. I relieved the constriction by gently insinuating the point of an aneurysm needle along the inguinal canal between the bowel and the external abdominal ring. When this was done and the anterior wall of the canal was gently raised, it was easy to return the gut into the abdominal cavity without any division of the tissues. I completed the operation by removing the sac and closing the ring: the wound healed by first intention.

But the result of a strangulated hernia in a baby is not always so satisfactory as in the cases just related. I have now under my care at the Victoria Hospital for Children, Chelsea, a boy, aged thirteen months, whose mother had noticed a reducible swelling in his right groin for three months. The child began to cry on January 27 at 6 P.M., and the mother said that the swelling then got bigger, and would no longer "go back." At 12.30 the same night the child began to vomit with great straining, and the vomit was yellow in colour. The bowels were last open on the morning of the 26th, and the mother felt sure that the child had not passed any flatus during the night. The child was brought to the hospital late on the afternoon of the 27th, and I operated for the relief of a strangulated hernia at six o'clock the same day. The sac contained a loop of empty small intestine, shrunken to a mere cord, and tightly constricted at the neck. I divided the constriction and reduced the hernia without much trouble. The child did well, and an enema given early on the morning of the 28th resulted in the passage of a satisfactory motion. At 2 P.M. the child suddenly became collapsed. It began to vomit, and by 8 P.M. the abdomen had become distended, and the peristalsis of the intestine was visible through the abdominal walls. I reopened the wound at 9 P.M. and drew out the loop of gut which had been strangulated. It had sloughed, and as the condition of the child seemed too bad to allow of any prolonged operation, I contented myself with drawing the loop well out of the wound, which I then packed lightly with gauze. The child was very ill for a few days, but succeeded in making a satisfactory artificial anus without any general peritonitis, thanks to the unremitting attention of Mr. Stanley Gibson, my house surgeon, and of the sister and nurses in the ward.

These cases show that if strangulated hernia is rare in very young children the prognosis is by no means bad: even the youngest patient may recover from conditions which seem well nigh desperate. But there are two points to which I would draw your attention in connection with strangulated hernia in young children. The hernia may be so transparent, even when it is strangulated, that you may mistake the swelling for a hydrocele, and there is often a marked suppression of urine, so that you may direct your attention to the kidneys rather than to the rupture.

### The Radical Operation.

The reasons in favour of operating upon children with hernia far outweigh in my opinion those which are advanced against the operation for radical cure, and I would operate much earlier and much oftener in hospital cases than in the children of better class parents. When a hernia in a child ceases to descend, the bowel no longer enters the inguinal canal the sac of necessity remains with the structures forming the cord distributed over and in intimate connection with it. You all know that during an operation for radical cure the sac has to be actually dissected away from the spermatic cord. The sac, therefore, cannot be withdrawn into the abdomen in a hernia which has been cured. It either shrinks and becomes converted into little more than a fibrous cord, or it remains patent ready to receive the bowel should it again become protruded through the abdominal ring. If it shrinks, the shrinkage may be complete and through its entire length; it may shrink above and below, leaving the middle portion unclosed. This may become distended with fluid, and an encysted hydrocele is the result; or it may close in the upper portion and become distended below when a hydrocele of the hernial sac is formed. The existence of the shrivelled sac, even in a cured hernia, seems to menace a recurrence. There is in all probability a shallow funnel-shaped depression of the peritoneum at the internal ring, which readily allows a fresh sac to be formed. An examination of the internal and external pillars of the abdominal ring show that one or the other is defective. It is not surprising, therefore, that a certain number of herniæ which seem to have been cured in childhood recur later in life, and often attain a large size in quite a short time. I operated upon such a case only last week. The patient, aged 22, was ruptured as a child, but was apparently cured. A hernia appeared suddenly, and painlessly, a few months before I saw him, and very quickly assumed a large size, filling and distending his scrotum. Such a recurrence may take place at a most inconvenient time, just before a physical examination for the public services, or when the boy has obtained a position which may prevent him from obtaining the leave of absence necessary to secure a satisfactory result after a radical operation. I recommend that an operation should be performed, therefore, in every case in which the hernia is not cured by the ordinary developmental changes taking place within a few months of birth, when by the application of a truss the child has been put into the best possible state to benefit by these changes. I would also advocate an operation where the abdominal wall remains manifestly weak in a child who has been congenitally ruptured, especially when the rings remain large and patulous, and the cord is thicker on the side which has been affected than upon the opposite side.

I prefer to operate when the child is between three and four years old, though I have no hesitation in recommending an operation much earlier if the hernia cannot be kept up by a truss: if it becomes strangulated or irreducible, or if the mother or nurse is incompetent.

### Hernia with Undescended Testicle.

I have dealt so far with ordinary inguinal herniæ in children, but there is another group which should not be omitted if the story is to be made complete. These are the herniæ associated with malposition of the testicle. These herniæ occur in two forms—in the one the rupture lies in the inguinal canal with an undescended testis, in the other the relation is less intimate between the testicle and the hernia. The hernia lies in a sac above, below, or between the muscles of the abdominal wall, and is then known as interstitial. The treatment of a hernia with an undescended testicle varies necessarily with the position of the testicle. When the testis is situated at the external ring or in the canal I always attempt to place it in the scrotum by freeing the cord as far as possible from the surrounding tissues and passing a suture through the scrotum and the testicle, the pillars of the ring being sutured. The scrotum often becomes invaginated, but the testicle is thereby retained outside the canal. When the testicle lies high up in the inguinal canal, and when it cannot be brought beyond the external abdominal ring, I do not hesitate to remove it, and afterwards close the canal in the same way as in the radical operation in a female. In any case, the existence of an undescended testicle seems to me to be an additional reason for performing a radical operation as soon as I have assured myself that there is no chance of the testicle assuming its proper position.

Interstitial herniæ form a very interesting group. They occur congenitally in boys, and as acquired herniæ in women. The hernia lies between the layers of the abdominal wall and above the level of the internal ring. The cause of the hernia is not definitely known, but it seems to be due to a weakness or faulty development in one part of the abdominal wall, whilst the region of the inguinal canal and external ring is normally formed; in other words, it is due to the predisposing causes of hernia acting at a higher point than usual. In boys, therefore, an interstitial hernia is usually associated with an undescended testis, whilst in women it occurs later in life in much the same way as an umbilical hernia. Some of you will remember this boy W. M., aged 13, who was in Henry Ward at the end of 1904, for I showed him at consultations as a good example of an interstitial hernia. He had a lump in the right groin which was clearly an undescended testicle, and a hernia situated one inch above Poupart's ligament, and one inch internal to the anterior superior spine of the ilium. The scrotum, as is usual in these cases, was undistended on the side of the hernia, but it was normally developed. The hernia lay between the external and internal oblique muscles, and I was able to perform a very satisfactory operation. The testicle was brought down into the scrotum and anchored there; the bowel was returned to the abdominal cavity, the sac was removed, and the gap in the muscles was closed with sutures. The boy has remained well, and, as you see, his hernia remains cured, and the abdominal wall is without any impulse when he coughs. The testicle lies indeed wholly outside the external abdominal ring, but it cannot be said to occupy the scrotum.

These, gentlemen, are some of the points to which I wished to draw your attention in connection with herniæ in children. You will find that they are worth bearing in mind, because although the surgery of children is a p[illegible]t of general surgery, it has it own differences and its own limitations.

